# *skn-1* is required for interneuron sensory integration and foraging behavior in *Caenorhabditis elegans*

**DOI:** 10.1371/journal.pone.0176798

**Published:** 2017-05-01

**Authors:** Mark A. Wilson, Wendy B. Iser, Tae Gen Son, Anne Logie, Joao V. Cabral-Costa, Mark P. Mattson, Simonetta Camandola

**Affiliations:** 1 Laboratory of Neurosciences, National Institute on Aging, Intramural Research Program, Baltimore, Maryland, United States of America; 2 Department of Experimental Radiation, Research Center, Dongnam Institute of Radiological and Medical Science, Jwadong-ri, Jangan-eup, Gijang-gun, Busan, Republic of Korea; 3 Department of Pharmacology, Institute of Biomedical Science, University of São Paulo, São Paulo, Brazil; 4 Department of Neuroscience, Johns Hopkins University School of Medicine, Baltimore, Maryland, United States of America; McGill University, CANADA

## Abstract

Nrf2/*skn-1*, a transcription factor known to mediate adaptive responses of cells to stress, also regulates energy metabolism in response to changes in nutrient availability. The ability to locate food sources depends upon chemosensation. Here we show that Nrf2/*skn-1* is expressed in olfactory interneurons, and is required for proper integration of multiple food-related sensory cues in *Caenorhabditis elegans*. Compared to wild type worms, *skn-1* mutants fail to perceive that food density is limiting, and display altered chemo- and thermotactic responses. These behavioral deficits are associated with aberrant AIY interneuron morphology and migration in *skn-1* mutants. Both *skn-1*-dependent AIY autonomous and non-autonomous mechanisms regulate the neural circuitry underlying multisensory integration of environmental cues related to energy acquisition.

## Introduction

The nuclear factor erythroid 2-related factor 2 (Nrf2) is a member of the Cap'n'Collar basic leucine zipper family known to play a key role in the cellular antioxidant and detoxification responses to stress [1]. In mammals under homeostatic conditions Nrf2 is maintained inactive via interaction with kelch-like ECH associated protein 1 (Keap1) and Cullin3. Cullin3 ubiquitinates and targets Nrf2 for continuous proteasomal degradation. In response to oxidative or electrophilic agents the interaction of Nrf2 with Keap1 is disrupted preventing Cullin 3-dependent degradation. The stabilized Nrf2 protein translocates into the nucleus where it dimerizes with small musculoaponeurotic fibrosarcoma (Maf) proteins to drive the transcription of cytoprotective genes [1]. Nrf2 and its invertebrate orthologs *skn-1* (*Caenorhabditis elegans*) and *CncC* (*Drosophila melanogaster*) have also been shown to promote health span and longevity. In *C*. *elegans* and *D*. *melanogaster* the genetic activation of the Nrf2 signaling pathway leads to enhanced longevity [2,3]. The comparison of Nrf2 activity in rodents with different maximum lifespan potential revealed a positive correlation between Nrf2 activation status and longevity [4]. Interestingly, despite showing high constitutive levels of active Nrf2 [4], the naturally long lived rodent naked mole rat has very low antioxidant enzyme expression [5]. This suggests that Nrf2 may exert pro-longevity benefits beyond its well characterize role as an antioxidant modulator. One of the best studied examples of biological conditions able to promote longevity in a wide range of experimental models is decreased nutrient availability [6, 7]. Caloric restriction and signaling pathways involved in metabolic homeostasis are recognized as universal effectors of longevity phenotypes [6–8]. Optimal metabolic homeostasis depends upon proper integration of intrinsic and extrinsic environmental signals by the nervous system. The impact of sensory integration (i.e. gustatory, olfactory, thermosensory) in health span and aging phenotypes has been extensively studied in invertebrates models [9]. For example, experimental impairment of olfaction extends lifespan in flies [10]. In *C*. *elegans*, mutations or manipulations impairing sensory neuronal function extend lifespan [11,12], and the longevity phenotype induced by caloric restriction depends upon the activity of Nrf2/*skn-1* in the ASI chemosensory neurons [13].

Activation of Nrf2 protects neurons in experimental models of stroke [14–16], Parkinson’s disease [17,18], and Huntington’s disease [19]. However, the contribution of the Nrf2 pathway to neuronal functions under physiological conditions is still largely unknown. In the present study we provide evidence that Nrf2 ortholog *skn-1* plays a fundamental role in foraging and food-related sensory integration in *C*. *elegans*.

## Material and methods

### Mice

C57BL/6 mice were breed and housed at the National Institute on Aging facility. Male (3 months old) were euthanized by carbon dioxide inhalation, and brains were removed and either dissected for immunoblot analysis, or processed for immunohistochemistry. All procedures were approved by the Institutional Care and Use Committee (IACUC) of the National institute on Aging (ASP 290-LNS-2019), and complied with the National Institutes of Health Guide for the Care and Use of Laboratory Animals.

### Western blot

Tissue extracts and western blot analysis were performed as described previously [14]. The primary antibodies used in this study were: Nrf2 (Cat: EP1808Y, Epitomics, Burlingame, CA), glyceraldehyde-3-phosphate dehydrogenase (GAPDH) (Cat: 2-RGM2, Advanced Immunochemical Inc, Long Beach, CA), glial-fibrillary acidic protein (GFAP) (GA5) (Cat: MAB360, EMD Millipore).

### Immunohistochemistry

Brains were fixed in 4% paraformaldehyde in PBS for 24 hours, then transferred to 30% sucrose in PBS at 4°C. Following antigen retrieval, sagittal sections were incubated for 1 h in blocking solution and then incubated overnight at 4°C with Nrf2 and glial fibrillary acidic protein (GFAP) antibodies. After extensive washing, the primary antibodies were detected using Alexa Flour 488- and 633-conjugated secondary antibody. Nonspecific labeling was determined by omission of the primary antibody. The cell nuclei were counterstained with DAPI (4',6'-diamidino-2-phenylindole) dye. Confocal images were acquired using a Zeiss 510 LSM microscope with 10X and 40X objective lenses.

### *Caenorhabditis elegans* strains

Worms were maintained on NGM agar plates with *E*. *coli* OP50 as food at room temperature (23°C) according to standard protocols [20]. Strains N2 Bristol wild-type strain, EU31 *skn-1(zu135)/DnT1)* putative *skn-1* null, EU1 *skn-1(zu67)/DnT1) skn-1a/c* null, FK134 *ttx-3(ks5)* putative *ttx-3* null, OH1098 *otIS133[ttx-3*::RFP + *unc-4(+)]*, OH2246 *otIS107[ser-2*::GFP] and OP342 *unc-119(ed3);wgIS342[skn-1*::TY1::EGFP::3xFLAG + *unc-119(+)]* were obtained from the Caenorhabditis Genetics Center at the University of Minnesota. OH1098 spontaneous males were crossed to OP342, and RFP/GFP double positive progeny bred to homozygosity to produce *wgIS342;otIS133* double transgenic worms. N2 males were crossed to OH2246, and F1 GFP+ males were then crossed to EU1. GFP+ F1 unc/het progeny were then crossed to *zu135/+* males, and resultant unc/het GFP+ progeny singled and selected for presence of the *zu135* allele, then bred to homozygosity for *otIS107* to introduce *ser2promoter1*::*GFP* into the *skn-1(zu135)* background.

### Transgenics

Transgenic strains were generated by microinjection. For all lines, 50 ng/μl of each expression construct was injected into N2 worms. Transgenes were crossed into the *skn-1* background as described above. *skn-1b* coding sequence was cloned from N2 young adult total RNA by rtPCR. 5kb *ttx-3* and 1kb *ric-19* promoter fragments were cloned from N2 DNA by PCR. Promoter and *skn-1b* insert fragments were cloned into pPD95.75 GFP expression vector [21]. The *mCherry*::*unc-54utr* fragment of pCFJ104 [22] was used to replace *gfp*::*unc54utr* of pPD95.75, then the 5kb *ttx-3* promoter fragment was inserted to create *pttx-3*::*mCherry* AIY specific marker CY691*(zu135/dnT1;bvEx177(pttx-3*::*mcherry;pric19*::*skn-1b*::*gfp))*. CY696*(zu135/dnT1;bvEx181 (pttx-3*::*mcherry;pttx-3*::*skn-1b*::*gfp))*. Primers are listed in S1 Table. PCR fragments were sequence verified.

### Behavioral assays

Chemotaxis assays were performed on 10 cm plastic petri dishes containing 10 ml assay medium (1.6% agar, 1mM CaCl_2_, 1mM MgSO_4_, 4mM NH_4_Cl, 25mM KH_2_PO_4_ pH 6.0), and analyzed as previously described [23]. Trials were performed in triplicate, with 25 animals per plate, unless otherwise indicated. Briefly, worms were transferred from culture plates to an empty assay plate and allowed to crawl freely for 10 minutes to remove residual bacteria. Worms were transferred to the center 1 cm of the assay plate (origin) and incubated at room temperature for 1 hour, at which time their positions were scored immediately. For sodium chloride assays, 5 μl of 5M NaCl was spotted onto one edge of the assay plate and left for 16 hours at room temperature to form a gradient prior to use [24]. Chemotaxis index was calculated as (O-S)/(T) where O was the number of animals at the odorant location and S the number of animals at the solvent control location after 1 hour and T the total number of worms. Thermotaxis assays were performed on radial thermal gradients on chemotaxis assay plates [25]. For dwelling assays, a 10 μl spot of diluted OP50 was spotted onto the center of the assay plate and allowed to dry for 1 hour. OP50 bacteria were grown overnight at 37°C with shaking, then washed twice with M9 buffer and diluted to an absorbance at A600 nm of 1.5 (1x concentration).

### *C*. *elegans* microscopy

Z-stack photomicrographs were acquired and analyzed on a Zeiss LSM510 inverted confocal microscope with LSM5 software. All images were taken using a 63x oil immersion objective with differential interference contrast. For quantification, identical areas of the worms were selected, and the total fluorescence was calculated as the mean fluorescence per μm^2^.

### Gene expression

Semi-quantitative rtPCR was performed on a Chromo4 system with Opticon 3 software (BioRad). Fold change was calculated using the ΔΔCt model [26], normalized to actin (*act-1*). Primers are listed in S1 Table.

## Results and discussion

**Fig 1:**
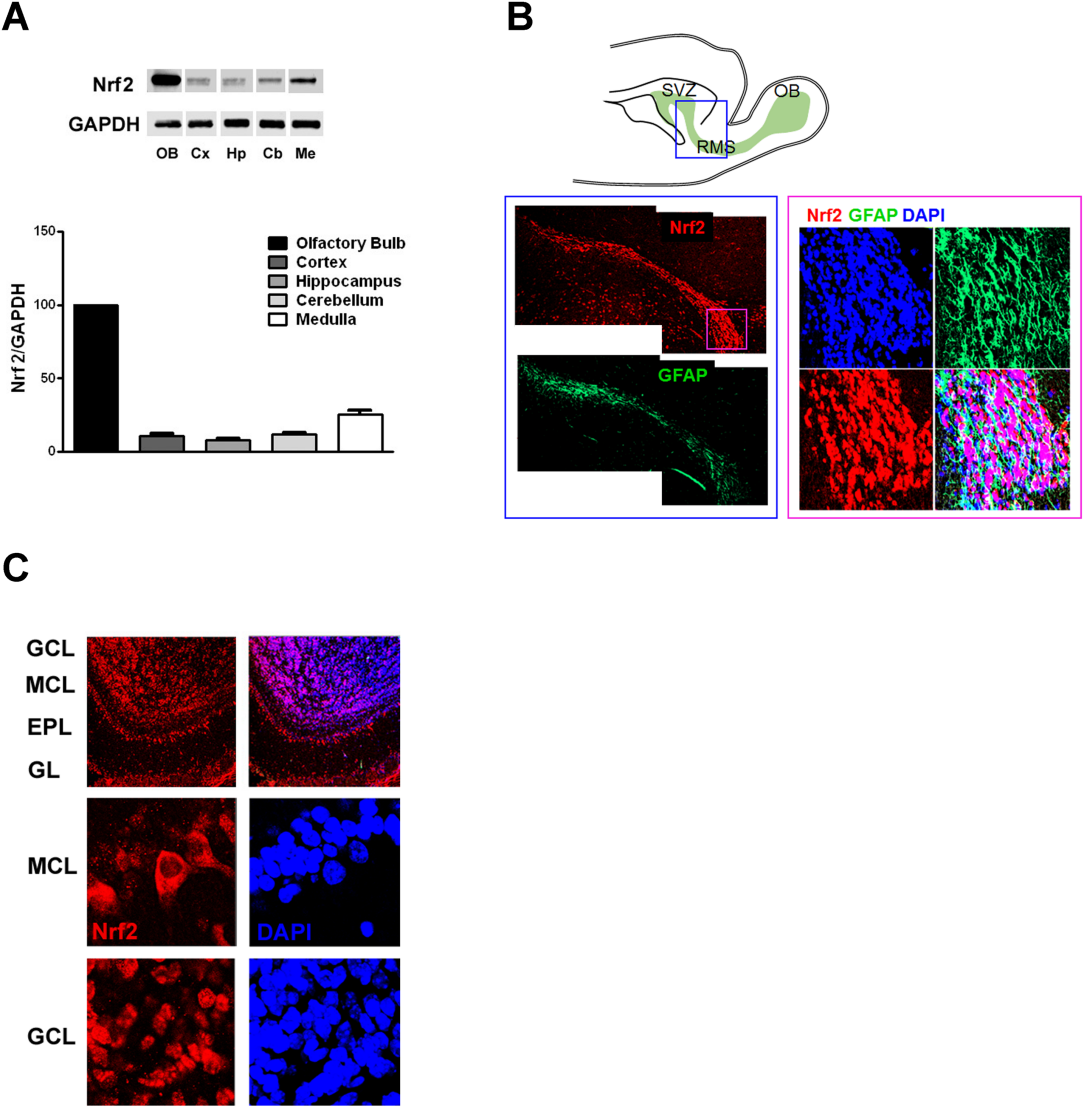
Nrf2 is highly expressed in olfactory bulb interneurons. (A) Representative immunoblots and quantification of Nrf2 protein levels in adult murine brain (n = 4). Values were normalized by GAPDH and expressed as mean percentage (and S.E.M.) compared to olfactory bulb (OB). Cx, cortex; Hp, hippocampus; Cb, cerebellum; Me, medulla. (B) Schematic showing the rostral migratory stream (RMS), the route followed by neuroblasts originating in the sub ventricular zone (SVZ) to reach the olfactory bulb (OB). Immunohistochemistry showing the expression of Nrf2 in the RMS at low magnification (left) and high magnification (right). The boxed area indicates the regions shown in the immunostaining. (C) Immunohistochemistry showing the distribution of Nrf2 in the various regions of the olfactory bulb (upper panel). GCL: granule cell layer; MCL: mitral cell layer; EPL: external plexiform layer; GL: glomerular layer. The higher magnification panels show Nrf2 subcellular localization in mitral cells (MCL) and granule cells (GCL).

In order to gain insights into the potential roles for Nrf2 in brain physiology, we first analyzed the levels of Nrf2 in five different regions of the mouse brain (olfactory bulb, cerebral cortex, hippocampus, cerebellum, and medulla). Immunoblot analysis revealed that Nrf2 protein levels in the olfactory bulb were four- to ten-fold greater than in any other brain regions evaluated (Fig 1A). Histological analysis of sagittal brain sections revealed high levels of Nrf2 expression in the rostral migratory stream (RSM), as well as in the mitral cell layer (MCL), and granule cell layer (GCL) of the olfactory bulb (Fig 1B and 1C). The RMS is a specialized pathway through which neuronal precursor cells originating in the subventricular zone migrate into the olfactory bulb (Fig 1B). Our findings of high Nrf2 expression in RMS cells substantiates recent evidence showing that in mice Nrf2 ablation and age-dependent alterations of the Nrf2 pathway, impair subventricular zone neuronal progenitor cell proliferation and survival [27–29]. Once the newly generated cells reach the olfactory bulb they differentiate into *γ*-aminobutyric acid (GABA) interneurons that integrate into the granule cell layer (GCL), or replace periglomerular cells in the glomerular layer (GL). In the olfactory bulb in addition to specific regional distribution we also observed distinct subcellular patterns of expression. In mitral cells Nrf2 is mainly located in the cytoplasm (Fig 1C), but in granule cell interneurons it is mostly concentrated in the nucleus, suggesting a constitutively active status in these cells (Fig 1C).

**Fig 2:**
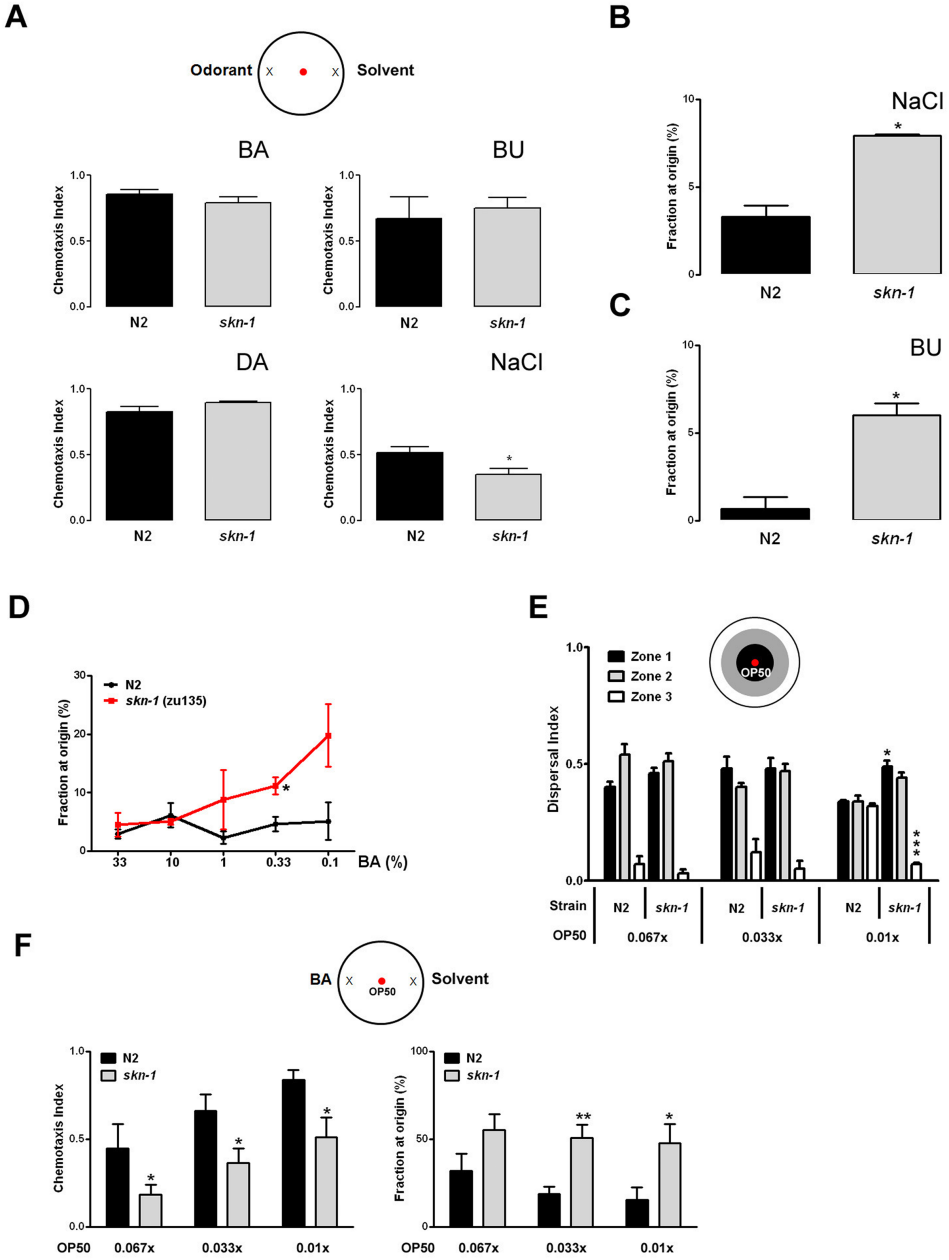
*skn-1* regulates sensory integration in *C*. *elegans*. (A) *skn-1(zu135)* worms showed wild type chemotaxis to optimal doses of volatile compounds and reduced migration to sodium chloride. Chemotaxis indexes were determined at the following odorant concentrations: 1% benzaldehyde (BA); 0.1% butanone (BU); 1% diacetyl (DA). Sodium chloride chemotaxis was tested on a 5 mM gradient. The percentages of animals remaining at the origin in presence of NaCl (B), butanone (C), or the indicated concentrations of benzaldehyde (D), are shown. (E) *skn-1* food-leaving behavior is impaired in the presence of very low local concentrations of food (OP50 *E*. *coli*). (F) *skn-1* chemotaxis toward BA is significantly decreased and dwelling behavior enhanced in the presence of known concentrations of bacterial lawn. Data are mean and S.E.M. of 3–6 trials performed in triplicate. *p<0.05; **p<0.01; ***p<0.001 versus N2 (Student’s *t*-test).

Because of its prominence in olfactory neurons we asked whether Nrf2 plays roles in chemosensation-related behaviors that enable animals to locate food sources and mates, as well as to avoid potentially harmful substances and predators. As the predominant sensory input, chemosensation is particularly important in the nematode *C*. *elegans*, where it regulates several metabolic and behavioral responses including pharyngeal pumping, locomotion, egg laying, life span and dauer formation [30]. In *C*. *elegans* the ortholog of Nrf2, *skn-1*, encodes three major isoforms (a, b and c) which each play distinct functional roles. *skn-1a* has been recently shown to localize to the mitochondrial membrane and respond to starvation [31]. *skn-1b* is expressed in neurons and mediates dietary restriction-induced longevity [13]. *skn-1c* on the other hand is intestinal and has been linked to oxidative stress resistance and longevity [2, 32]. To assess the role of *skn-1*/Nrf2 in chemosensory perception we took advantage of the fact that the *C*. *elegans* nervous system and behavioral responses to odorants are well-characterized. We performed standard chemotaxis experiments comparing the ability of wild type N2 and *skn-1(zu135)* null worms to respond to known chemoattractants. There were no significant differences in the chemotaxis index for volatile compounds such as benzaldehyde (BA) (Fig 2A and S2 Table), butanone (BU) and diacetyl (DA) which are recognized by AWC and AWA sensory neurons. However, we did find a small significant decrease in ASE-dependent chemotaxis index toward sodium chloride in *skn-1* worms (Fig 2A). During the course of our chemotaxis experiments we noted a tendency of *skn-1* worms to remain in close proximity to the origin of the assay plate. When we analyzed this behavior in more detail, we found that a significant fraction of *skn-1* worms preferred to remain in the center area of the plate regardless of the type of the compound, soluble or volatile, used as chemoattractant (Fig 2B and 2C). This behavior was more pronounced when suboptimal concentrations of chemoactractant were used (Fig 2D and S3 Table), did not depend upon the *skn-1* background as the *skn-1(zu67)* strain showed a similar phenotype (S1A Fig and S3 Table), and was not related to impaired motility as demonstrated by identical dispersal indexes (S1B Fig).

In addition to salt chemoattraction, ASE neurons have recently been shown to promote adaptive food-leaving behavior as food becomes limiting [33]. Chemotaxis enables nematodes to locate new food sources, and many chemoattractants are products of bacterial decay. One explanation for the behavioral phenotype we observed is that *skn-1* worms can detect very small amounts of carried over bacteria and prefer to dwell around the origin area. To test this possibility we performed ‘leaving behavior’ assays in which known dilutions of OP50 were placed at the origin. As shown in Fig 2E at concentrations that N2 worms considered indicative of no or negligible levels of food, and led them to roam throughout the plate, *skn-1* worms preferred to remain in the area close to the bacterial lawn. Because of this enhanced sensitivity to very small quantities of bacteria, it is possible that for *skn-1* worms the chemotaxis experiments performed under standard conditions were actually multi-sensory integration experiments. We thus decided to compare the behavior of N2 and *skn-1* worms in conditions where an attractant and a specific concentration of OP50 *E*. *coli* placed at the origin were provided as sensory cues. We found that in such experimental settings *skn-1* worms preferred to dwell on the OP50 lawn rather than move toward the chemoattractant (Fig 2F and S4 and S5 Tables). These results suggest that *skn-1* worm behavior under conditions requiring integration of multiple food-related sensory cues is impaired compared to wild type animals.

**Fig 3:**
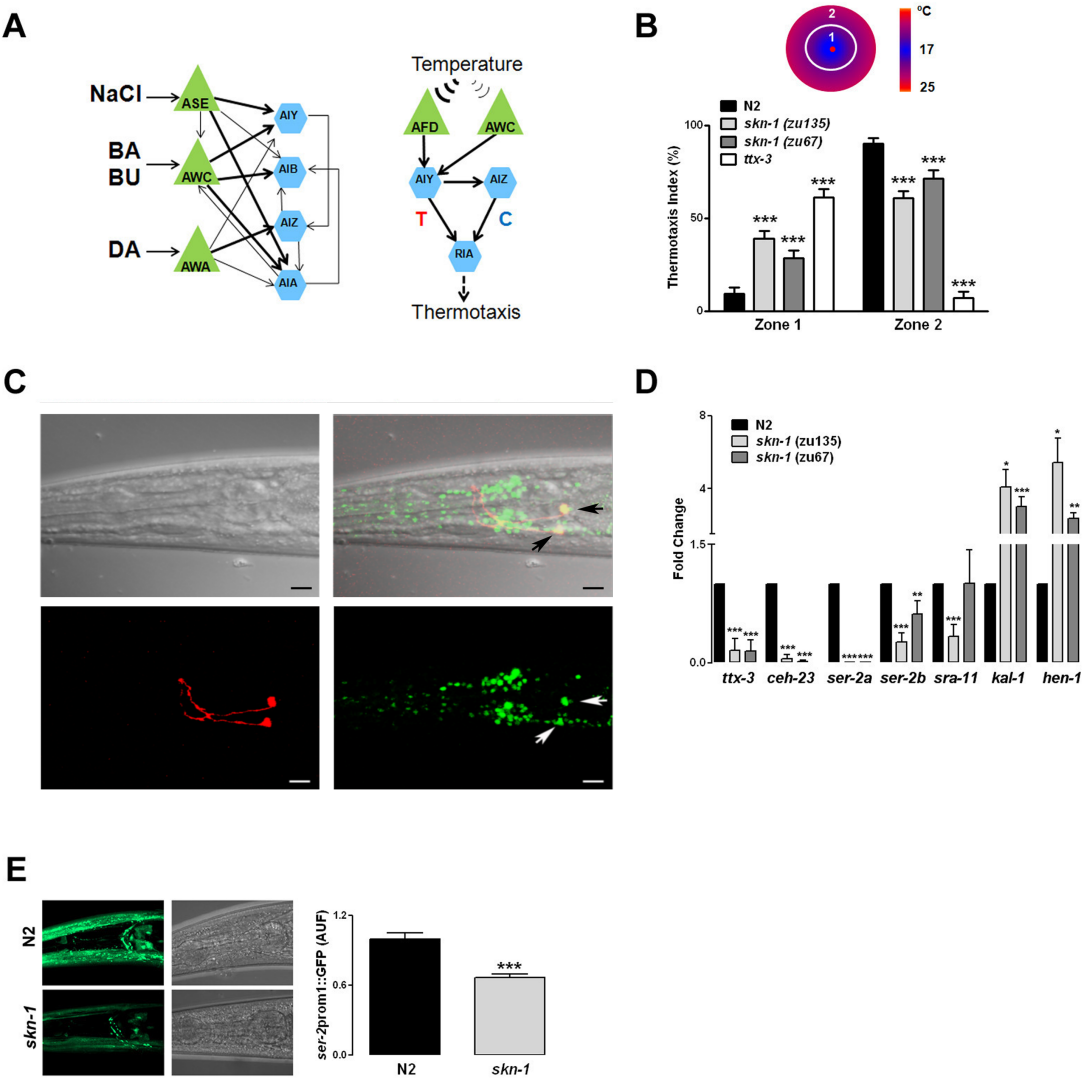
*skn-1* is expressed in AIY neurons and regulates their functions. (A) Schematic of the neuronal circuits of *C*. *elegans* chemoattractive and thermotactic behaviors. Green triangles represent sensory neurons and blue hexagons command interneurons. Arrows indicate direct interactions, and their thickness is proportional to the frequency of synaptic contacts between the neurons (Adapted from [36]). T, thermophilic; C, cryophilic.(Adapted from [41]). (B) Population thermotaxis experiments were performed using a radial thermal gradient. After 60 minutes the position of the worms was scored and the percentages of worms in the colder (Zone 1) and warmer (Zone 2) regions determined. N2 animals moved toward the region of the plate closer to their previous cultivation temperature (23°C), while both *skn-1* null strains *zu135* and *zu67* showed a cryophilic phenotype similar to *ttx-3* (AIY) null worms. (C) Representative 3D reconstruction of confocal sections through the head region of double transgenic *wglS342;otlS133* worms is shown. The *skn-1*:*EGFP* reporter (green), containing the entire *skn-1* gene, is highly expressed in neurons in the head ganglia, and colocalizes with the AIY-specific marker *ttx3*:RFP (Red). Arrows point at AIY. Scale bar 10 μm. (D) Relative mRNA levels of the AIY cell fate specification homeobox genes *ttx-3* and *ceh-23*, and their targets *ser-2a*, *ser-2b*, *sra-11*, *kal-1* and *hen-1* in N2 and *skn-1* null worms. (E) Representative 3D reconstructions showing the expression of the transcriptional reporter *ser2*:*prom1*:*EGFP* in the head region of N2 and *skn-1(zu135)* null worms. Data are mean and S.E.M. of 3–5 experiments. *p<0.05; **p<0.01; ***p<0.001 versus N2 (Student’s *t*-test).

Interneurons play a central role in the integration of different sensory modalities. Based on the distinct distribution of Nrf2 in the mammalian olfactory interneurons, the perturbed chemotaxis behavior in *skn-1* mutant *C*. *elegans* and the known *C*. *elegans* neuronal wiring (Fig 3A), we focused on AIY and AIA as interneurons potentially responsible for the observed *skn-1* behavioral phenotype. Previous findings suggest that AIY interneurons are critical for chemotaxis responses to near-threshold levels of odors [34], food choice behavior [35] and dwelling [34–36]. We took advantage of the fact that AIY interneurons are essential for *C*. *elegans* thermotaxis behavior through AFD sensory neurons (Fig 3A) [37]. We hypothesized that if AIY functions are altered in *skn-1* worms, they will likely display abnormal thermotaxis behavior. The ability of N2 and *skn-1* worms to migrate toward their cultivation temperature (23°C) was assessed in population thermotaxis assays using *ttx-3* mutant worms in which AIY neurons are dysfunctional [38–40]. While N2 animals moved toward the region of the plate closer to their previous cultivation temperature, both *skn-1* loss-of-function strains *zu135* and *zu67* showed a cryophilic phenotype similar to, although not as pronounced as *ttx-3* null worms (Fig 3B and S6 Table). Overall our behavioral results indicate that *skn-1* is required for proper integratory functions of sensory interneurons. Notably, altered olfactory integration with impaired fine discrimination has also recently been shown in Nrf2 knock out mice [29].

With regard to the *C*. *elegans* nervous system, *skn-1* has been shown to be expressed in ASI [13] and dopaminergic neurons [42]. Because our data suggest that *skn-1* is required for normal AIY function, we analyzed *wgIS342;otIS133* double transgenic animals using confocal microscopy. Because of the large genomic DNA sequence used to create the construct, *wglS342* drives the expression of all *skn-1* isoforms under endogenous coding and regulatory sequences, thus providing a more comprehensive and faithful expression pattern. As shown in Fig 3C, *skn-1*::EGFP is highly expressed in neurons in the lateral, ventral and dorsal ganglia, and to a lesser extent in the anterior ganglia. Furthermore, *skn-1*::EGFP clearly colocalizes with the specific AIY marker *ttx-3*::RFP, confirming that *skn-1* is indeed expressed in AIY interneurons.

**Fig 4:**
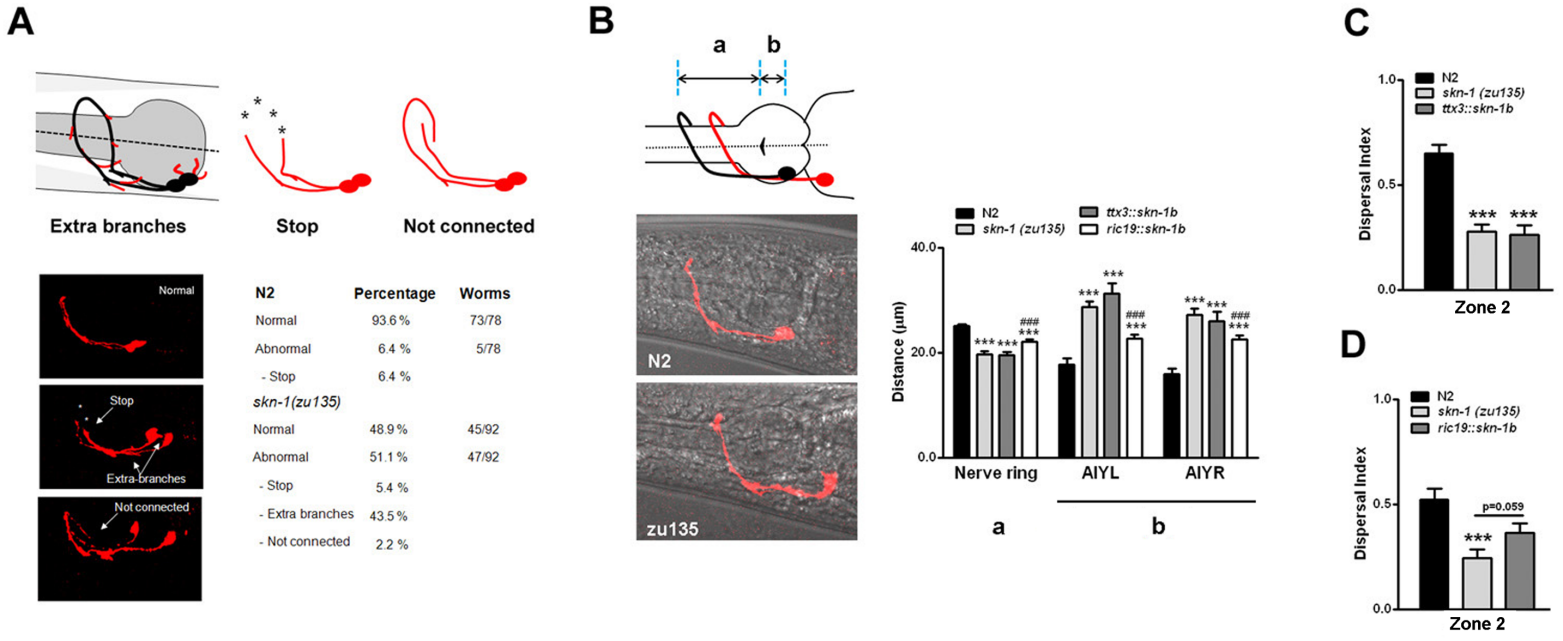
*skn-1* regulates AIY neuron morphology and food-seeking behavior by cell-autonomous and non-autonomous mechanisms. (A) Schematic depiction, representative images, and quantification of the neuroanatomical defects observed in *skn-1(zu135)* worms. Wild type morphology is shown in black, while aberrant branches from the axon or cell body are depicted in red. Also shown are examples of premature stops, and axon misrouting. (B) Schematic depiction and representative images of the AIY position relative to the pharynx grinder in N2 (black line) and *skn-1(zu135)* worms (red line). a, distance between grinder and the AIY axon fasciculation at the nerve ring; b, distance between the grinder and AIY cell body. Quantification of the AIY migratory defects in the indicated worm strains. AIY-specific expression of *skn-1b* has no effect on migration (B), and food-leaving behavior (C) (OP50 = 0.033x). Pan-neuronal expression of *skn-1b* significantly decreases AIY migratory defects (B) and partially rescues the food-leaving behavior (D). Data are mean and S.E.M. of 3–7 experiments. ***p<0.001 versus N2; ###p<0.001 versus *skn-1* (zu135) (Student’s *t*-test).

The homeodomain transcription factors *ceh-10*, *ttx-3* and *ceh-23* coordinate the AIY cell fate specification [43]. We used qRT-PCR to quantify the expression of these transcription factors and some of their known targets in N2 and *skn-1* worms. Levels *of ceh-10* were comparable amongst the various strains (data not shown), however both *ttx-3* and *ceh-23* were significantly reduced in *skn-1(zu135)* and *(zu67)* worms (Fig 3D). *ser-2a* and *ser-2b*, which encode a tyramine receptor, were also significantly reduced in *skn-1* worms (Fig 3D). *skn-1(zu135)*, but not *skn-1(zu67)*, showed decreased levels of *sra-11* (Fig 3D), suggesting a specific effect of the *skn-1b* isoform in the regulation of this G protein-coupled receptor implicated in olfactory imprinting [44]. We verified the changes in *ser-2* levels by analyzing the expression pattern of GFP driven by *ser2*::*prom1* in N2 and *skn-1(zu135)* transgenic animals. As shown in Fig 3E a significant decrease for *ser-2*:: GFP expression was observed in neurons as well as pharyngeal cells and head muscles in *skn-1* worms. Surprisingly, levels of *hen-1* and *kal-1* were increased in both *skn-1* strains (Fig 3D). In humans Kallmann syndrome is characterized by hypogonadism, and hyposmia or anosmia. In *C*. *elegans* misregulation of the Kallmann syndrome gene ortholog *kal-1* has been shown to alter neurite branching and the formation of axon collaterals, as well as axon pathfinding, and establishment of target connections [45,46]. The analysis of AIY morphology in *skn-1* worms showed a significant increase of aberrant branching from the axon, as well as of short neurite-like projections from the cell body (Fig 4A). We also observed a less penetrant phenotype of axonal misrouting characterized by the axon of one of the AIY pair running over and around the dorsal midline, and failing to interact with the contralateral axon (Fig 4A). Such defects depended upon levels of *skn-1* expression, as heterozygous *skn-1* animals displayed an intermediate percentage of abnormalities (normal = 73.7%; abnormal = 26.3%). Furthermore, regardless of the presence of morphological defects we observed that, relative to the pharynx grinder, the position of the AIY cell bodies in *skn-1* worms was far more caudal compared to N2 worms (Fig 4B), while the distance of the AIY axon fasciculation at the nerve ring was shorter (Fig 4B). Similar neuroanatomical defects have been described in *ttx-3* mutant animals [42,46]. We assessed the possibility that our behavioral and morphological findings result from *skn-1* dependent misregulation of AIY by generating transgenic animals in which *skn-1b* cDNA is specifically and exclusively driven in AIY by a *ttx-3* promoter (CY696). Compared to *skn-1* mutants, CY696 animals displayed about 54% less AIY morphological defects (normal = 76.3%; abnormal = 23.7%), but identical AIY migratory (Fig 4B), and dwelling behavioral defects (Fig 4C and S7 Table). These findings suggest that *skn-1b* acts at least partially in a cell autonomous fashion to control AIY morphogenesis, whereas the AIY migratory and behavioral impairments are non-AIY autonomous defects.

Because *ttx-3* is normally expressed in other head neurons (AIA, ADL, ASI, ADF) [34,43], and the AIY specific expression of *skn-1b* is not sufficient to rescue the behavioral abnormalities, we tested a *ric-19* promoter to drive the pan-neuronal expression of *skn-1b* (CY691). CY691 transgenic animals had about 41% less AIY anatomical defects (normal = 69.9%; abnormal = 30.1%), and an intermediate phenotype between N2 and *skn-1* null worms in terms of AIY migratory defects (Fig 4B), and food-leaving behavior (Fig 4D and S8 Table). This result, together with the behavioral similarities we observed between the *skn-1* strains lacking all three *skn-1* isoforms (*zu135*) and *skn-1a/c* isoforms (*zu67*), suggest that expression of *skn-1b* in non-neuronal cell types is required for a full functional recovery.

Nrf2/*skn-1* is widely known for its role in adaptive responses to many different types of stress, particularly oxidative stress. Accordingly, much attention has been given to Nrf2/*skn-1* target genes that encode antioxidant proteins [47]. Studies of cell culture and animal models have shown that Nrf2/*skn-1* plays a major role in the process of hormesis, in which exposure of a cell or organism to a low level of stress (e.g., oxidative, metabolic, xenobiotic) results in resistance to greater levels of the same or different stressor [48]. Moreover, recent findings suggest that Nrf2/*skn-1* mediates, in part, beneficial effects of bioenergetics challenges such as food restriction on organ system function, disease resistance, and longevity [49–52]. Previous studies of *C*. *elegans* have shown that phytochemicals that activate Nrf2/*skn-1* can significantly increase lifespan [53], and that *skn-1* in ASI neurons is required for the lifespan-extending effect of food deprivation [13]. Our data reveal a previously unknown role for Nrf2/*skn-1* in regulating the organization and functionality of neuronal networks involved in behavioral responses of worms to food availability, temperature, and specific odorants. We speculate that this ‘decision-making’ role for *skn-1* in AIY neurons evolved to optimize adaptive behavioral responses to a wide range of environmental challenges faced by worms in their normal habitat.

## Conclusions

Our findings show that Nrf2/*skn-1* is expressed in interneurons involved in chemosensation in mice and *C*. *elegans*, and that genetic deletion of *skn-1* results in impaired sensory integration. The inability to activate *skn-1* signaling causes the *skn-1* worms to linger in inadequate food patches rather than migrate toward new potential food sources. Lack of *skn-1* results in morphological, migratory and functional alterations of AIY suggesting that *skn-1* is important for the proper development of a pair of neurons that play a pivotal role in coordinating behavioral responses to food and temperature, two fundamental environmental factors influencing growth, reproduction and survival.

## Supporting information

S1 Fig (A) Percentage of N2 and *skn-1(zu67)* animals remaining at the origin in presence of the indicated concentrations of benzaldehyde. (B) N2 and *skn-1(zu135)* worms display identical locomotion abilities. Animals were placed on an empty plate and allowed to crawl freely for 60 min. Their position was scored and the percentage of animals in the center (Zone 1) and periphery (Zone 2) determined. Data are mean and S.E.M. * p<0.05 versus N2.
(TIF)

S1 Table List of primers used for quantitative PCR and cloning. (TIF)

S2 Table Primary data for the analysis of chemotaxis response to benzaldehyde. (TIF)

S3 Table Primary data for the analysis of the fraction of animals remaining at the origin during chemotaxis experiments performed with the indicated concentration of benzaldehyde. (TIF)

S4 Table Primary data for the analysis of chemotaxis response to 1% benzaldehyde in presence of known concentrations of OP50 at the origin. (TIF)

S5 Table Primary data for the analysis of the fraction of animals remaining at the indicated concentration of OP50 (origin) during chemotaxis experiments performed in 1% benzaldehyde). (TIF)

S6 Table Primary data for the analysis of thermotaxis response in a radial thermal gradient. (TIF)

S7 Table Effect of skn-1b reconstitution in AIY interneurons on dwelling behavior performed in presence of 0.033x OP50 at the origin. (TIF)

S8 Table Effect of pan-neuronal skn-1b reconstitution on dwelling behavior performed in presence of 0.033x OP50 at the origin. (TIF)
