# Supplementary figures and images for: skn-1 is required for interneuron sensory integration and foraging behavior in Caenorhabditis elegans
Source: PLoS One. 2017 May 1;12(5):e0176798. doi: 10.1371/journal.pone.0176798 (PMC5411085; doi:10.1371/journal.pone.0176798)

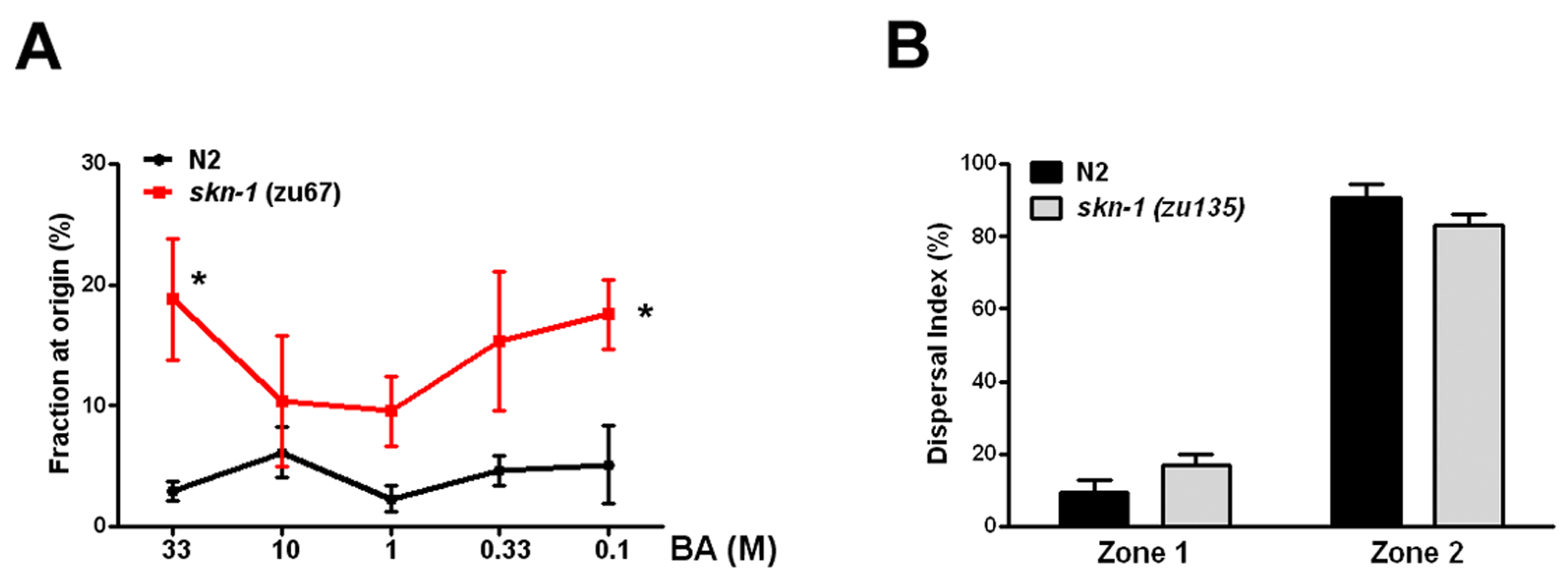

Supplement: S1 Fig — (A) Percentage of N2 and skn-1(zu67) animals remaining at the origin in presence of the indicated concentrations of benzaldehyde. (B) N2 and skn-1(zu135) worms display identical locomotion abilities. Animals were placed on an empty plate and allowed to crawl freely for 60 min. Their position was scored and the percentage of animals in the center (Zone 1) and periphery (Zone 2) determined. Data are mean and S.E.M. * p<0.05 versus N2. (TIF) [file pone.0176798.s001.tif]

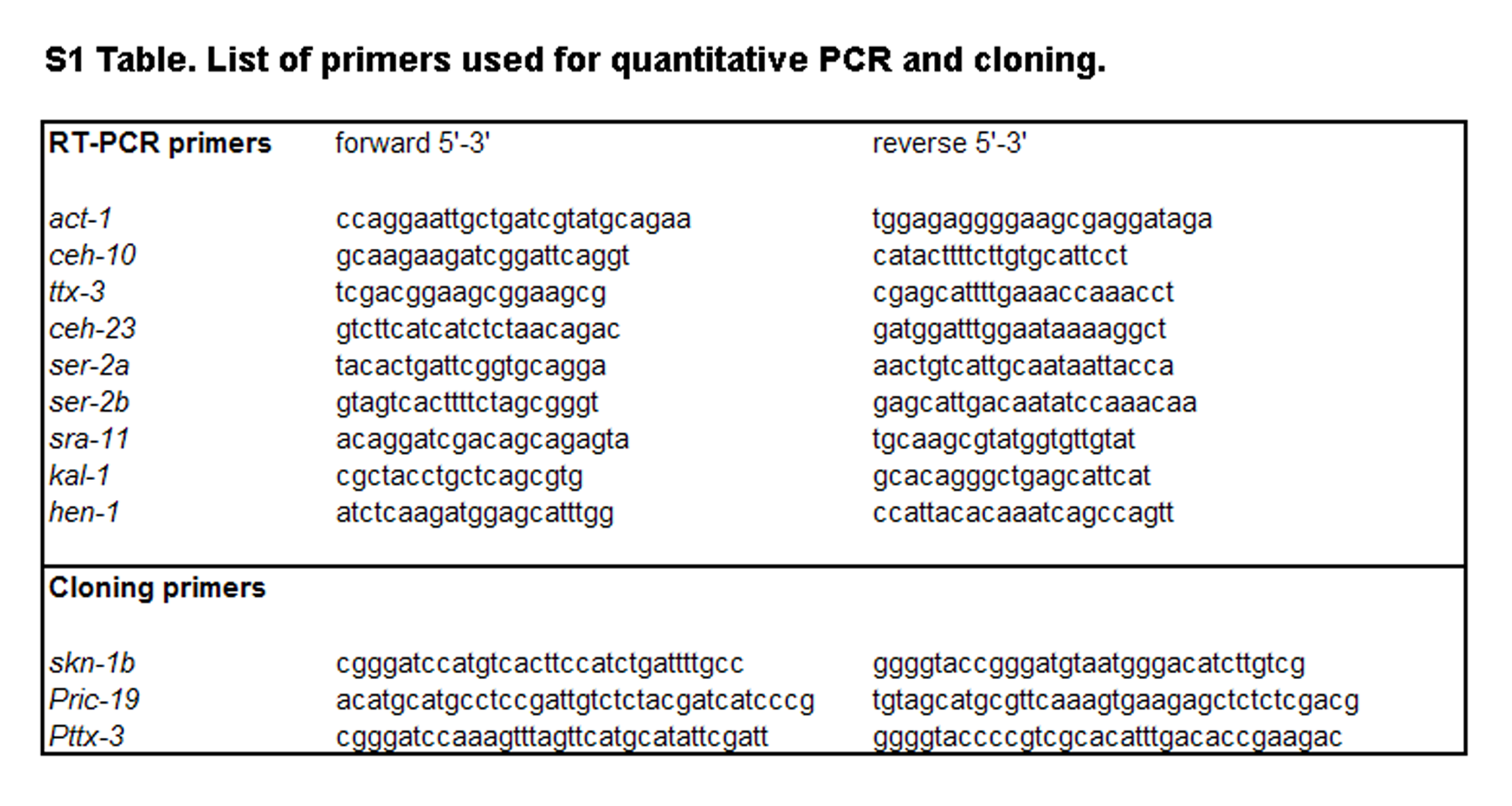

Supplement: S1 Table — (TIF) [file pone.0176798.s002.tif]

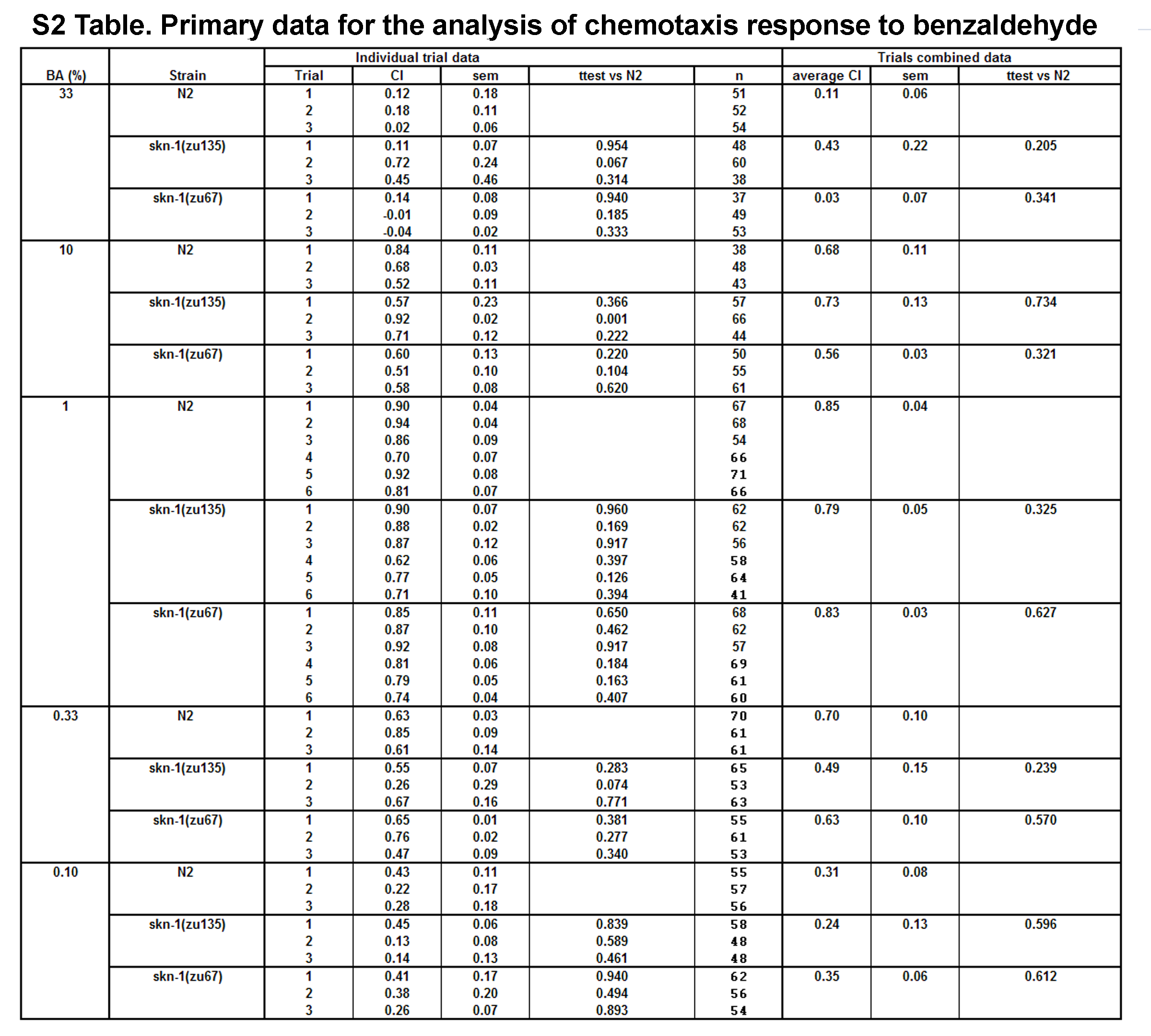

Supplement: S2 Table — (TIF) [file pone.0176798.s003.tif]

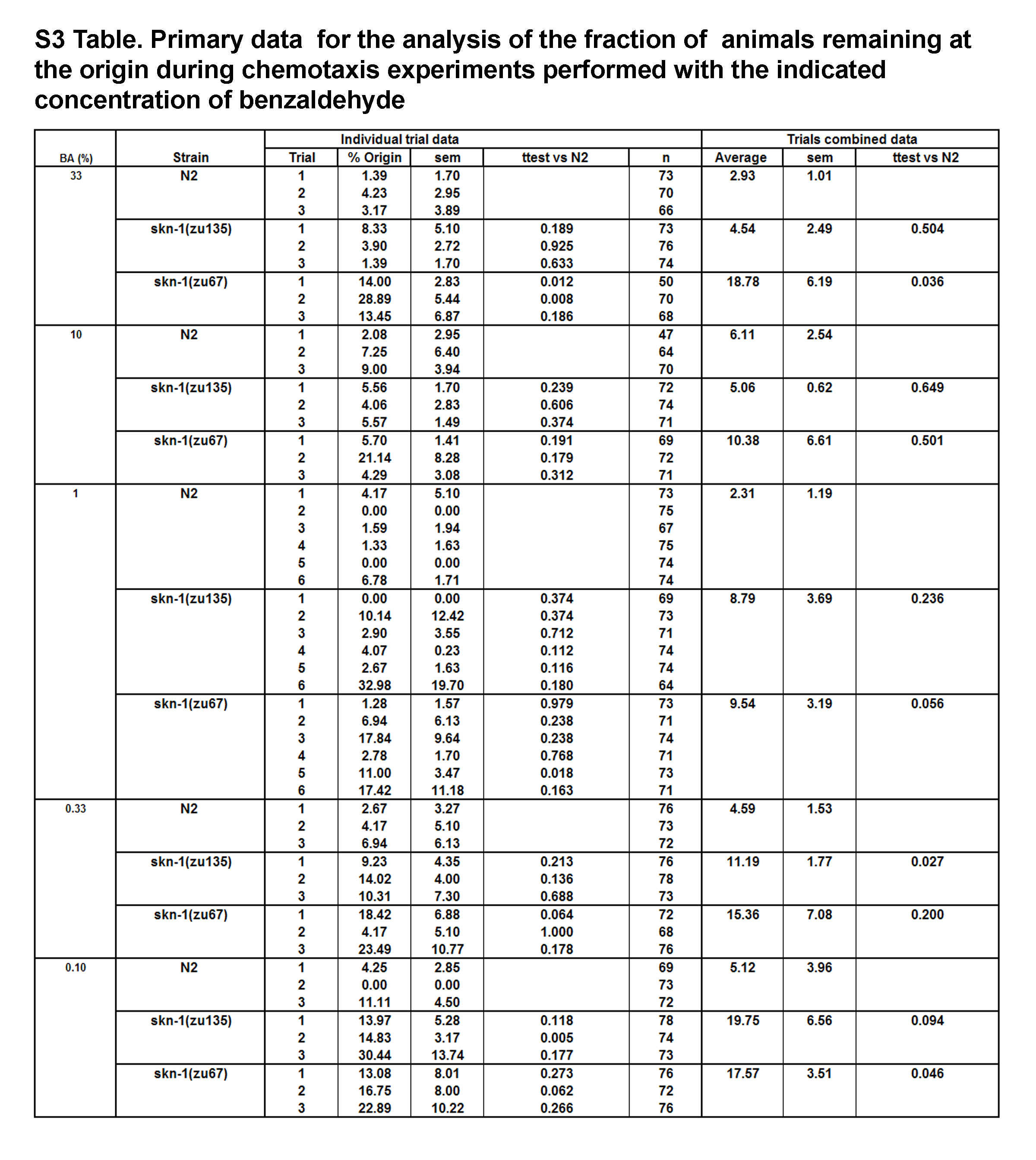

Supplement: S3 Table — (TIF) [file pone.0176798.s004.tif]

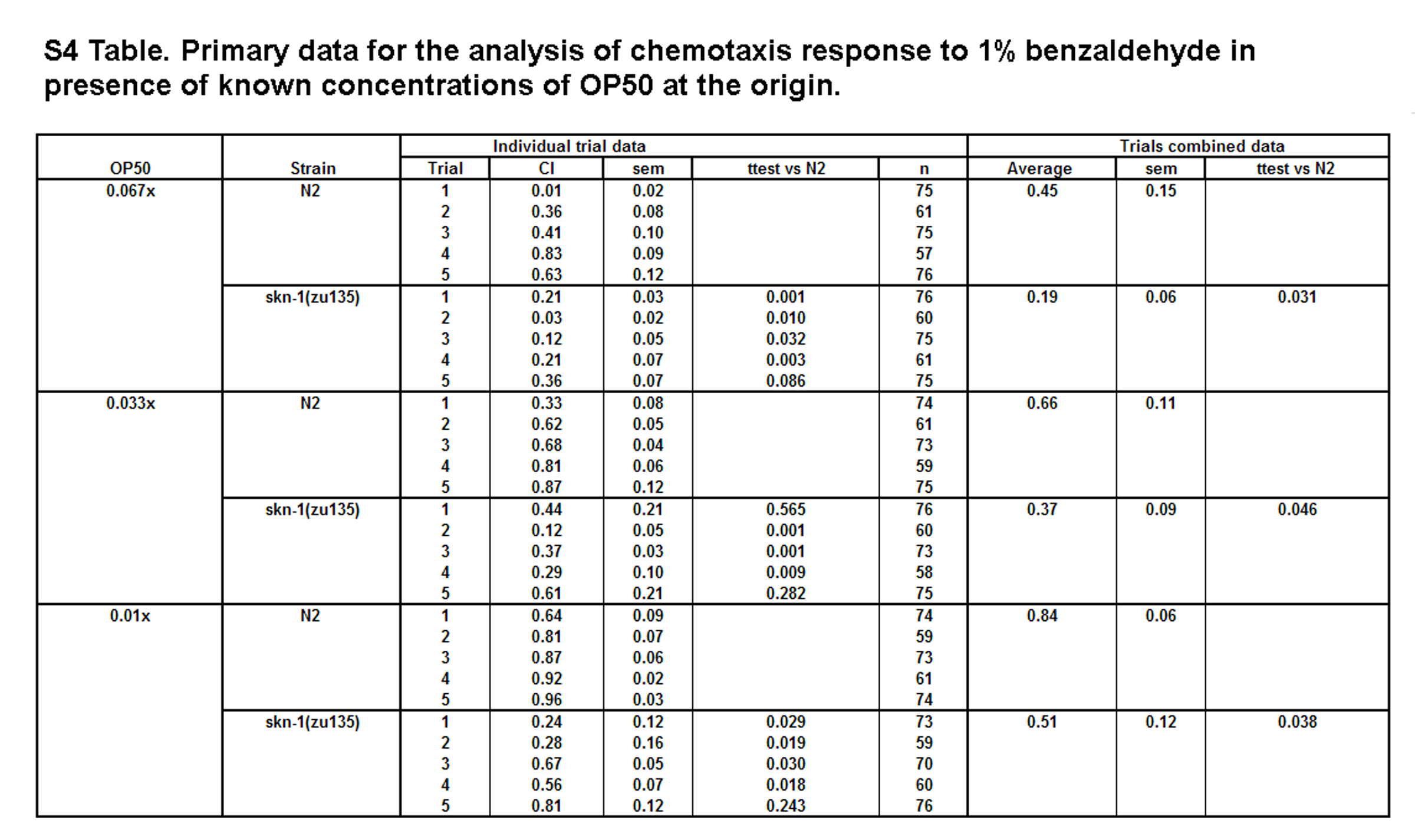

Supplement: S4 Table — (TIF) [file pone.0176798.s005.tif]

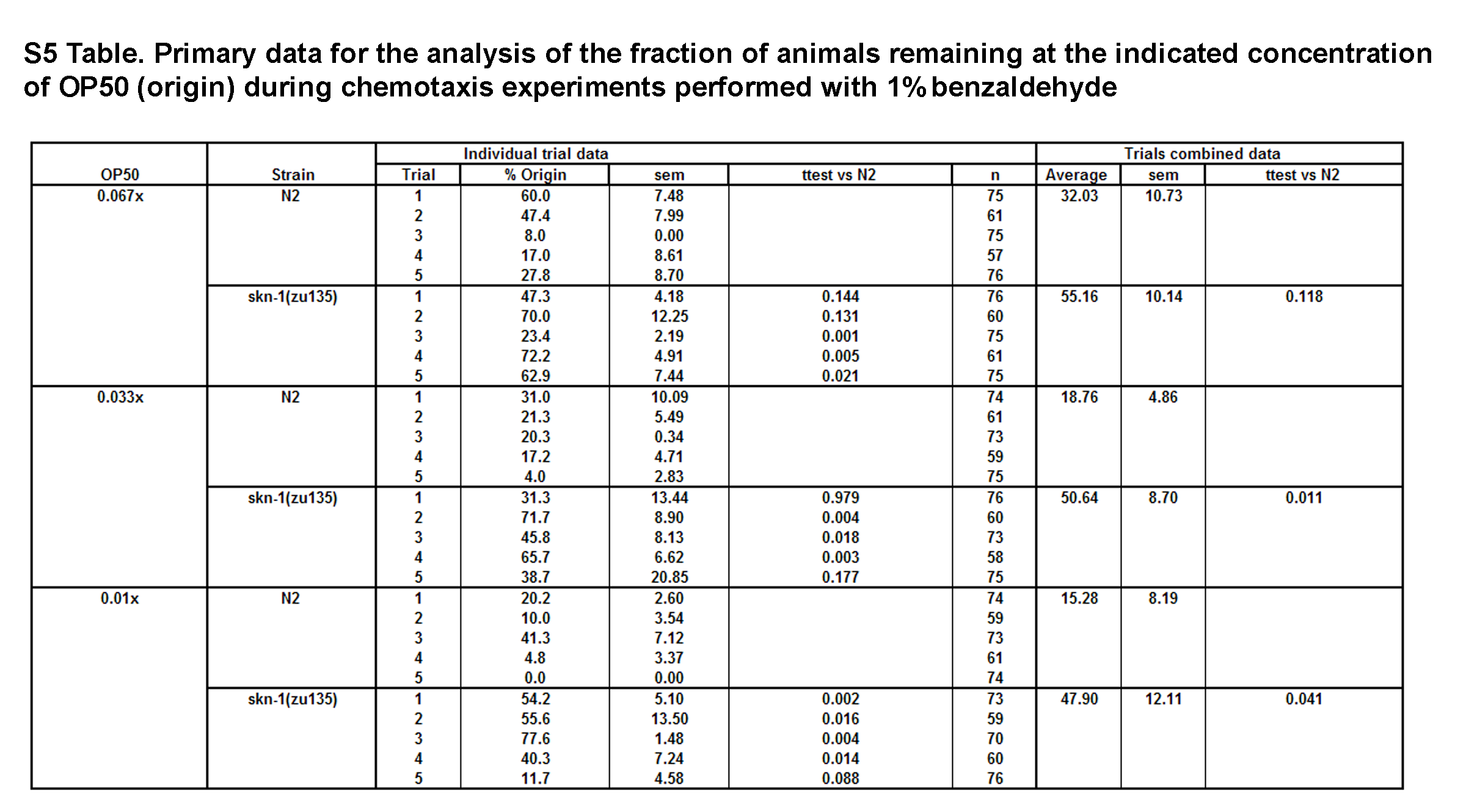

Supplement: S5 Table — (TIF) [file pone.0176798.s006.tif]

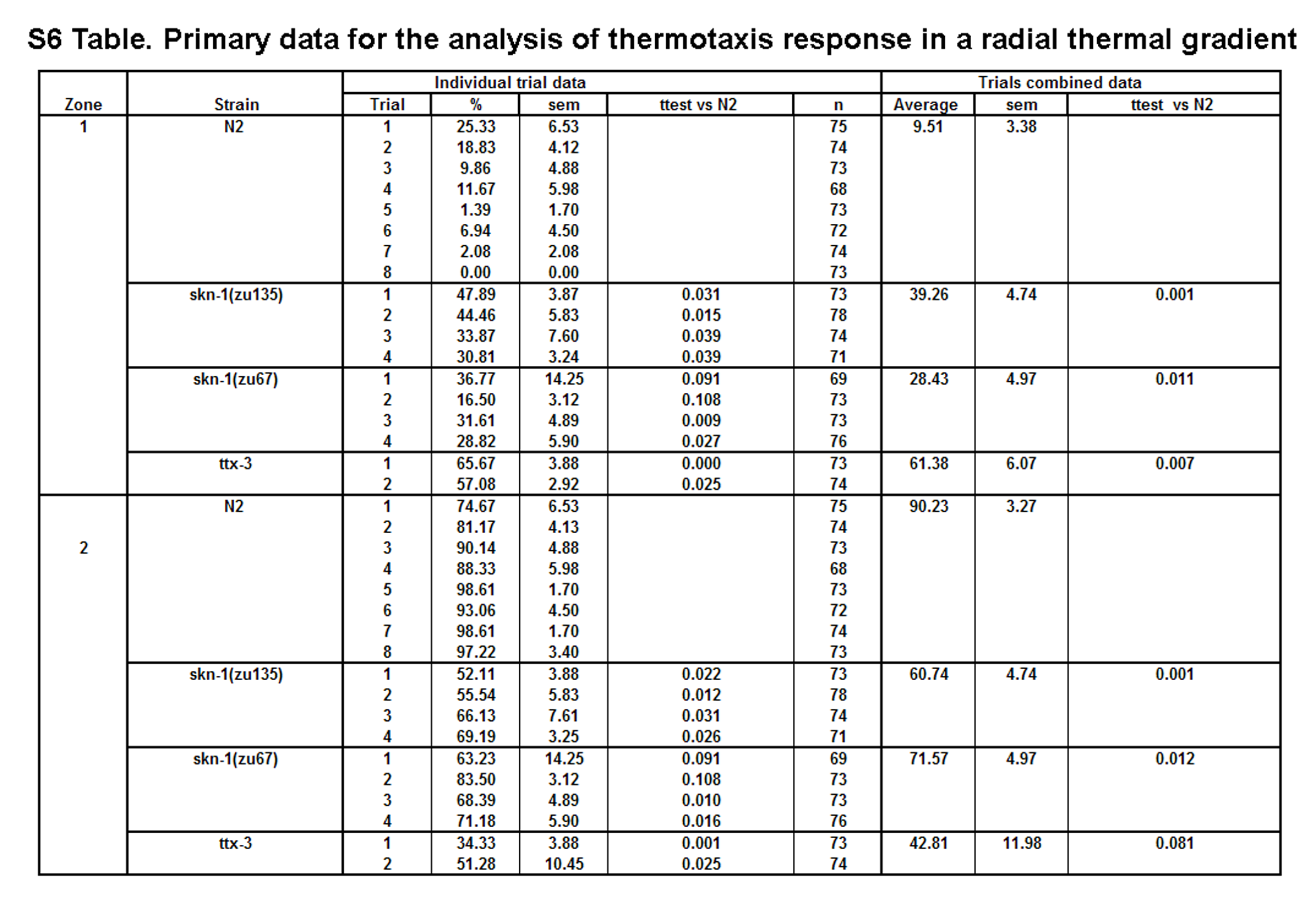

Supplement: S6 Table — (TIF) [file pone.0176798.s007.tif]

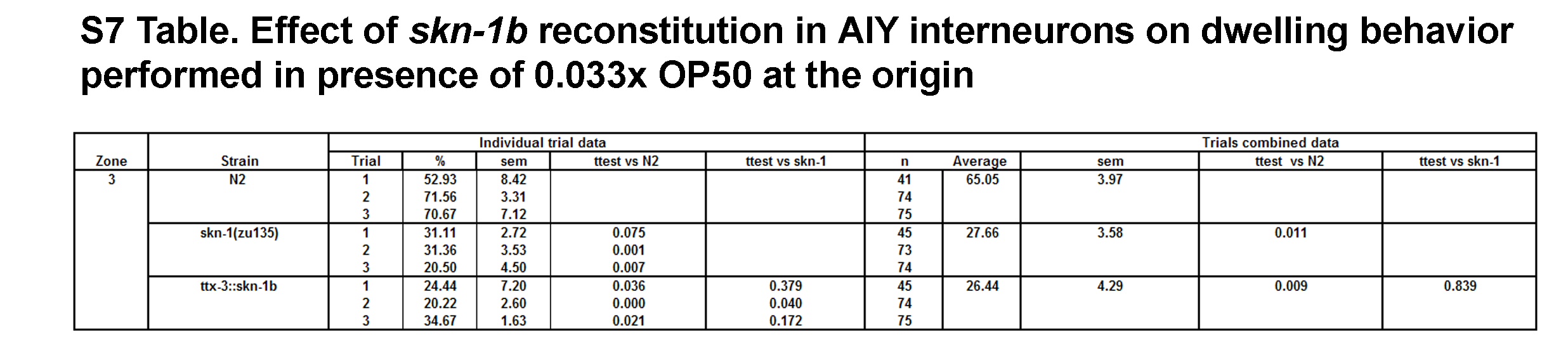

Supplement: S7 Table — (TIF) [file pone.0176798.s008.tif]

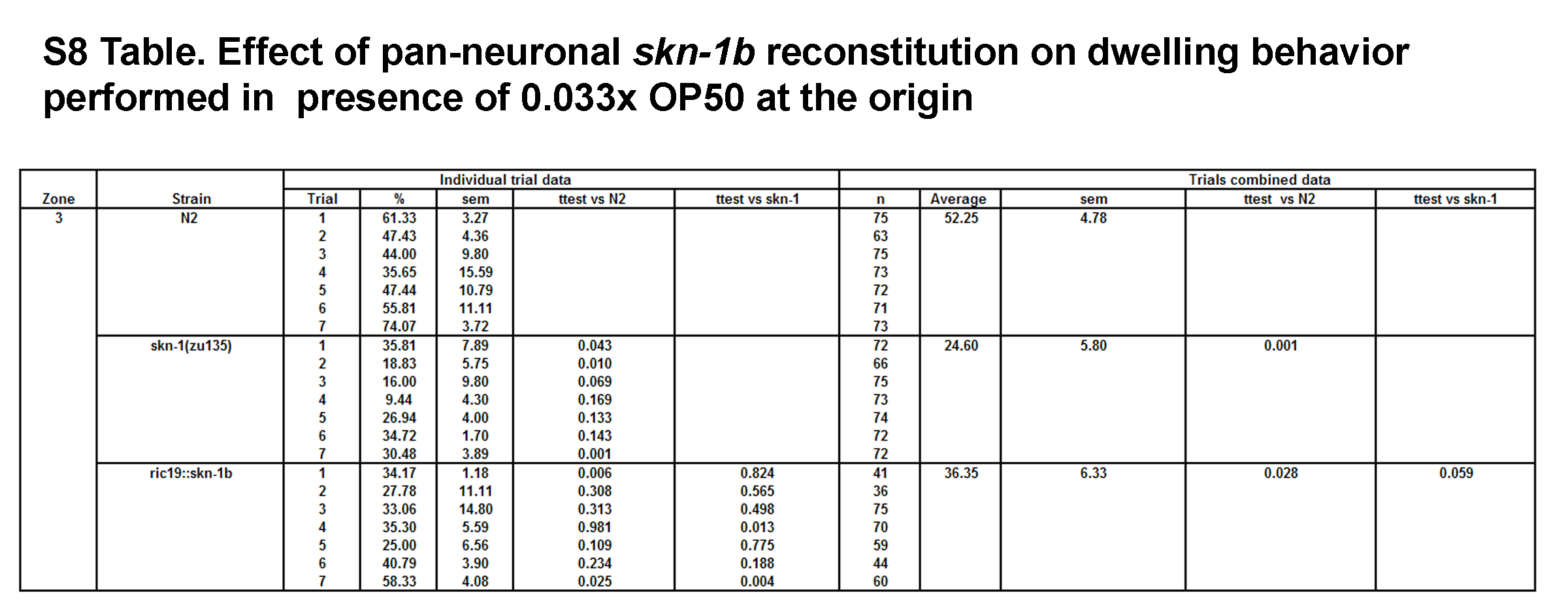

Supplement: S8 Table — (TIF) [file pone.0176798.s009.tif]
